# ADVANCE CARE PLANNING (ACP) CONCORDANCE ASSOCIATED WITH RELATIONSHIP QUALITY AND GRIEF AMONG DEMENTIA DYADS

**DOI:** 10.1093/geroni/igae098.1623

**Published:** 2024-12-31

**Authors:** Sara Bybee, Jordana Clayton, Nancy Aruscavage, Rebecca Utz, Eli Iacob, Kara Dassel

**Affiliations:** University of Utah College of Nursing, Salt Lake City, Utah, United States; University of Utah, Salt Lake City, Utah, United States; University of Utah, Salt Lake City, Utah, United States; University of Utah, Salt Lake City, Utah, United States; University of Utah, Salt Lake City, Utah, United States; University of Utah, Salt Lake Cty, Utah, United States

## Abstract

Advance care planning (ACP) is linked to enhanced physical and mental well-being, fewer unwanted hospitalizations, and improved quality of life for individuals and their care partners. We investigated the impact of the Life-Planning in Early Alzheimer’s and Other Dementias (LEAD) Guide on ACP concordance—the mutual understanding and agreement on ACP values and preferences—among persons living with dementia (PLWD) and their care partners, and its impact on relationship quality and grief. Dyads (N=48) participated in a discussion of each partner’s answers to the LEAD Guide, addressing any discrepant responses. Participants completed open-ended survey questions (n=43) regarding their: discussion, confidence in their partner’s understanding of their end-of-life wishes, confidence in their partner’s ability to make decisions aligning with their end-of-life wishes, and any additional comments. An interpretive descriptive approach to qualitative analysis revealed four themes: 1) Relationship quality between partners (high versus strained relationship quality, 2) Advance care planning process and discussion (ACP concordance versus discordance), 3) Engagement in ACP, and 4) The grief process associated with dementia disease trajectory. Dyads with more ACP concordance codes demonstrated more codes related to high relationship quality (versus strained relationship quality) and fewer grief codes. Conversely, dyads with more ACP discordance codes had more strained relationship quality and grief codes. Our findings suggest that the LEAD guide may enhance ACP concordance, contributing to improved relationship quality and reduced grief. Clinicians may consider additional support for dyads demonstrating greater ACP discordance given its co-occurrence with strained relationship quality and grief.

